# The Role of Gibberellins and Brassinosteroids in Nodulation and Arbuscular Mycorrhizal Associations

**DOI:** 10.3389/fpls.2019.00269

**Published:** 2019-03-15

**Authors:** Peter N. McGuiness, James B. Reid, Eloise Foo

**Affiliations:** School of Natural Sciences, University of Tasmania, Hobart, TAS, Australia

**Keywords:** nodulation, arbuscular mycorrhizae, plant hormone, gibberellin, brassinosteroid, DELLA, symbioses

## Abstract

Plant hormones play key roles in nodulation and arbuscular mycorrhizal (AM) associations. These two agriculturally and ecologically important symbioses enable plants to gain access to nutrients, in particular, nitrogen in the case of nodulation and phosphorous in the case of AM. Work over the past few decades has revealed how symbioses with nitrogen-fixing rhizobia, restricted almost exclusively to legumes, evolved in part from ancient AM symbioses formed by more than 80% of land plants. Although overlapping, these symbiotic programs also have important differences, including the *de novo* development of a new organ found only in nodulation. One emerging area of research is the role of two plant hormone groups, the gibberellins (GAs) and brassinosteroids (BRs), in the development and maintenance of these symbioses. In this review, we compare and contrast the roles of these hormones in the two symbioses, including potential interactions with other hormones. This not only focuses on legumes, most of which can host both symbionts, but also examines the role of these in AM development in non-legumes. GA acts by suppressing DELLA, and this regulatory module acts to negatively influence both rhizobial and mycorrhizal infection but appears to promote nodule organogenesis. While an overall positive role for BRs in nodulation and AM has been suggested by studies using mutants disrupted in BR biosynthesis or response, application studies indicate that BR may play a more complex role in nodulation. Given the nature of these symbioses, with events regulated both spatially and temporally, future studies should examine in more detail how GAs and BRs may influence precise events during these symbioses, including interactions with other hormone groups.

## Introduction

While the application of fertilizer to crop plants may compensate for poor nutrient availability in the soil, there may be negative impacts such as the use of non-renewable resources (e.g., phosphate), cost, enhancing the growth of weeds, or runoff into waterways which may cause eutrophication. An alternative way to enhance growth under low nutrient conditions is by increasing plant nutrient uptake *via* plant-microbe symbioses with arbuscular mycorrhizal fungi or rhizobial bacteria. Arbuscular mycorrhizal symbioses are ancient and widespread, occurring in over 80% of land plant taxa (Smith and Smith, 2011). In these symbioses, the fungi act as an extension of the plant’s root system, increasing the surface area for nutrient uptake (Yang et al., 2015). In return, the plants provide the fungi with energy in the form of fatty acids and sugars (e.g., Smith and Smith, 2011; Yang et al., 2015; Jiang et al., 2017). Arbuscular mycorrhizal symbioses can increase plant growth and yield in soils poor in essential nutrients, particularly phosphorus (Baum et al., 2015; Yang et al., 2015). Another important plant microbe symbiosis almost completely restricted to legumes is nodulation (Mus et al., 2016), where nitrogen-fixing rhizobial bacteria are housed in nodules in the root system. The nitrogen-fixing bacteria convert atmospheric nitrogen into ammonia, which the plant can use as a nitrogen source (Mus et al., 2016). Nodulation is thought to have evolved in part from the ancient arbuscular mycorrhizal program (Delaux et al., 2013; Martin et al., 2017).

Both nodulation and arbuscular mycorrhizal symbioses are regulated by a variety of plant signals, including many of the plant hormones (Ferguson and Mathesius, 2014; Gutjahr, 2014; Bedini et al., 2018; Liao et al., 2018). This review focuses on how two families of plant growth hormones, the gibberellins (GAs) and brassinosteroids (BR), influence the development of rhizobial and AM symbioses. It is timely to synthesize an overview across species and to examine any parallels between the two symbioses. In particular, we examine the stage of the symbiosis at which the hormones operate, as these are complex processes with distinct infection and development phases that can occur across different spatial and temporal scales. Although interactions between GA and BR has been suggested to occur at the level of signaling (for review see Ross and Quittendon, 2016), this has not yet been explored during symbioses. Although there is evidence that some rhizobial bacteria may produce GA (Tatsukami et al., 2016; Nett et al., 2017), it is not yet clear if mycorrhizal fungi produce GA, as this has yet to be tested using modern quantitative techniques (e.g., Barea and Azcón-Aguilar, 1982). As the GAs and BRs also have powerful effects on many other aspects of plant development, for example, stem elongation (Ross et al., 2011), in this synthesis, we take care to examine the evidence to determine whether the effect of the hormones on a symbiosis is direct or may result from indirect effects of these hormones on other aspects of the plant’s phenotype.

## Gibberellins

GAs are a group of diterpenoid growth hormones strongly associated with promoting growth, including stem elongation and germination (e.g., Ross and Reid, 2010). The bioactive GAs signal by binding to intracellular receptors from the GID protein family, which then complex with DELLA transcription factors and an E3 ubiquitin ligase. The E3 ubiquitin ligase polyubiquitinates the DELLA proteins, tagging them for degradation (Daviere and Achard, 2013). DELLA proteins can act as represses of transcription; thus, the loss of the DELLA transcription factors in the presence of GA derepresses gene expression inducing GA response (Daviere and Achard, 2013).

### Gibberellins Regulate Arbuscular Mycorrhizae

**Figure 1:**
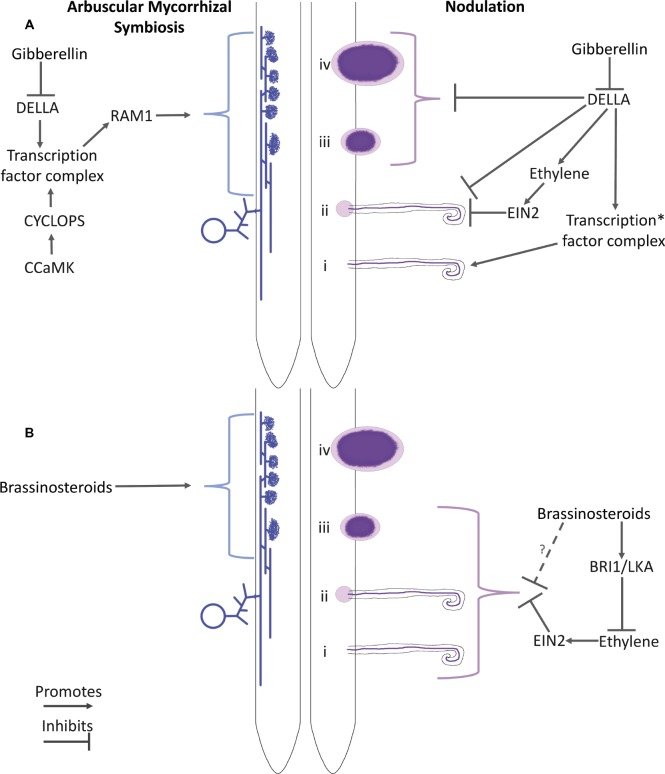
Schematic of the proposed roles of gibberellin **(A)** and brassinosteroids **(B)** and their interaction with ethylene in arbuscular mycorrhizal colonization (LHS) and nodulation (RHS). Key players in hormone perception and potential downstream elements are indicated. For arbuscular mycorrhizal colonization, a spore, hyphae, and arbuscules are represented. For nodulation, numerals indicate the stages of development: (i) Infection thread formation, (ii) initial cortical cell divisions, (iii) nodule organogenesis, and (iv) development of the nodule into a nitrogen-fixing organ. *Proteins that have been proposed to influence this transcription factor complex include CCaMK, IPD3, NSP2, and NF-YA1 (See the text for references).

There is good evidence that arbuscular mycorrhizal colonization is inhibited by GA signaling (Figure 1A). Several studies have shown that the application of GAs inhibits the formation of arbuscular mycorrhizae, including studies in pea (El Ghachtouli et al., 1996), *Lotus japonicus* (Takeda et al., 2015), and tomato (Martín-Rodríguez et al., 2015). A negative role for GAs in arbuscular mycorrhizal development was confirmed by mutant and transgenic studies in pea, *Medicago truncatula,* rice, and wheat (Floss et al., 2013; Foo et al., 2013; Yu et al., 2014). The severely GA-deficient pea mutant *na*, which has a non-functional *ent*-kaurenoic acid oxidase enzyme (Davidson et al., 2003), has more arbuscular mycorrhizae than wild-type plants (Foo et al., 2013). This influence of low gibberellins on mycorrhizal colonization appears to be independent of ethylene (Foo et al., 2016). However, it acts through the DELLA proteins since a *DELLA*-deficient double mutant of pea, *la cry-s,* which results in permanently high GA signaling, and the *na la cry-s* triple mutant display reduced levels of arbuscular mycorrhizal colonization compared with wild-type plants (Foo et al., 2013). The formation of arbuscular mycorrhizal symbiosis occurs in several stages, including spore germination and hyphal branching, hyphopodium formation, and penetration into the root, followed by the development of hyphae and branched arbuscules (Figure 1; Genre et al., 2005). GA signaling through DELLA appears to be particularly involved in arbuscule initiation, rather than arbuscule branching or hyphal colonization, as loss-of-function *della1 della2* double mutants in pea and *Medicago truncatula* and the *della* mutant *slr1* in rice display a more dramatic reduction in arbuscules than wild-type plants compared to other fungal structures (Floss et al., 2013; Foo et al., 2013; Yu et al., 2014). Consistent with this, a rice *SLR-YFP* overexpression line and a wheat *Rht1/Rht2* gain-of-function *DELLA* line displayed increased arbuscule formation (Floss et al., 2013; Yu et al., 2014). Although they are altered in number, the arbuscules that do form in plants with altered GA or DELLA status appear relatively normal (Floss et al., 2013; Foo et al., 2013).

The establishment of a symbiosis requires the recognition, uptake, and accommodation of the microbe. For both nodulation and arbuscular mycorrhizal associations, this is initiated by the plant’s perception of unique microbial-derived lipochitooligosaccharides or chitin oligomers, known as Nod and Myc factors, respectively (Martin et al., 2017). Genetic studies in legumes have begun to define the parts of this pathway that the two symbioses share, known as the common symbiotic signaling cascade, and the outputs unique to each symbiosis. One key output of the perception of the Nod and Myc factors is the induction of calcium spiking that is perceived by the CCaMK protein. In the formation of mycorrhizal symbioses, CCaMK phosphorylates the transcription factor CYCLOPS/IPD3, which activates a variety of symbiosis genes that facilitate fungal infection and arbuscule formation (Zipfel and Oldroyd, 2017). An important downstream target of this pathway is RAM1. RAM1 is a GRAS transcription factor required for arbuscule initiation and branching (Pimprikar et al., 2016). Several studies have examined how DELLA may influence elements of this mycorrhizal signaling cascade. Transactivation studies, yeast two hybrid studies, bimolecular fluorescence complementation and co-immunoprecipitation studies have revealed that DELLA proteins may participate in a complex with CYCLOPS (Jin et al., 2016) and CCaMK to control RAM1 expression (Pimprikar et al., 2016). Genetic evidence for such interactions during AM development are complex and are complicated by the fact that aspects may rely on AM activation of the cortical program by CCaMK (Floss et al., 2013). Overall, studies with mutants, transgenic lines, and application of GA or GA biosynthesis inhibitors are consistent with DELLA acting with CCaMK and CYCLOPS to promote arbuscule formation *via* RAM1 (Floss et al., 2013; Pimprikar et al., 2016). However, it is important to note that gain-of-function DELLA overexpression or GA biosynthesis inhibitors can restore arbuscule formation in *cyclops* mutants; this indicates the potential for other DELLA interacting partners (Pimprikar et al., 2016).

Another approach to examine the dynamics of GA during arbuscular mycorrhizal colonization was undertaken in a study in *Lotus japonicus*, in which transgenic plants expressing the *GUS* reporter gene under the control of promoters of the GA biosynthesis genes *GA20ox1* and *GA20ox2* were generated (Takeda et al., 2015). Co-staining for GUS and arbuscular mycorrhizae revealed cells expressing these GA biosynthesis genes corresponded to cells hosting the AM fungus (Takeda et al., 2015). Bioactive GA_1_ levels in whole infected roots were also somewhat elevated compared to uninoculated roots. Plants forming an arbuscular mycorrhizal symbiosis accumulate GAs in their roots, potentially due to increased activity in the 13-hydroxylation GA biosynthesis pathway (Martín-Rodríguez et al., 2015). In addition, the study by Takeda et al. (2015) proposed that exogenous GA influences fungal entry into the plant and hyphal branching of the mycorrhizal fungi, although as these studies were necessarily conducted *in planta*, it is difficult to distinguish direct effects on the fungi from indirect effects due to altered plant growth.

### Gibberellins Regulate Nodulation

GA can have both positive and negative effects of the number of nodules formed. Application of bioactive GAs and/or GA biosynthesis inhibitors can both promote and inhibit nodulation, depending on the species, dose, and application method (e.g., Thurber et al., 1958; Mes, 1959; Ferguson et al., 2005; Lievens et al., 2005; Fonouni-Farde et al., 2016). This is consistent with genetic studies, where low nodule numbers are observed in pea, *Medicago*, and *Lotus* mutants with high GA signaling, due to *della* mutations, and also in pea mutants with low GA signaling, due to disruption of GA biosynthesis (Ferguson et al., 2005; Fonouni-Farde et al., 2016; Jin et al., 2016; McAdam et al., 2018).

While this apparent paradox may be explained by stating that there is an optimal level of GA for nodulation overall, this may disguise the fact that GA exerts different effects on specific stages of nodulation (Figure 1B; Ferguson et al., 2005; Ferguson et al., 2011; McAdam et al., 2018). As outlined above, nodulation is induced by Nod factor signaling *via* the common symbiotic pathway. This signaling not only facilitates infection at the epidermis but also induces concomitant induction of nodule organogenesis in the root cortex. These initially spatially separated events can occur independently, as demonstrated by the development of spontaneous nodules in the absence of rhizobia in gain-of-function CCaMK or cytokinin receptor mutants (e.g., Marsh et al., 2007; Tirichine et al., 2007). GA appears to have a strong negative effect on the infection stage of nodulation since loss-of-function *della* mutants or transgenic lines in *Medicago* and pea display a reduced number of infection threads compared with wild-type plants (Fonouni-Farde et al., 2016; Jin et al., 2016; McAdam et al., 2018). Consistent with this, infection thread formation is also suppressed by exogenous GA in a range of species (Maekawa et al., 2009; Fonouni-Farde et al., 2016; Jin et al., 2016; McAdam et al., 2018). Similarly, GA-deficient mutants of pea display elevated infection thread formation (McAdam et al., 2018). Yeast three hybrid and co-immunoprecipitation studies in *Medicago* suggest that this negative influence of GA signaling on infection may be mediated through physical interaction of the DELLA proteins with key components of the Nod factor signaling pathway including NF-YA1, NSP1, NSP2, and IPD3/CYCLOPS (Fonouni-Farde et al., 2016; Jin et al., 2016). DELLAs have also been reported to increase the phosphorylation state of IPD3/CYCLOPS *in vitro* (Fonouni-Farde et al., 2016). Consistent with this, Nod factor-activated gene expression was suppressed by GA treatment in wild-type *Lotus* and *Medicago* and, in the absence of GA treatment, in *Medicago della* mutant lines (Maekawa et al., 2009; Fonouni-Farde et al., 2016; Jin et al., 2016).

Several different approaches have been used to examine the role of GA during nodule initiation, organogenesis, and ultimate function. In addition to displaying reduced infection, lines with loss of DELLA function also display a reduced number of nodules (Ferguson et al., 2011; Fonouni-Farde et al., 2016; Jin et al., 2016; McAdam et al., 2018). However, the nodules that do form on *della* mutants appear similar to wild type and in pea appear to fix nitrogen at a similar rate to wild-type nodules (McAdam et al., 2018). This suppression of nodule initiation in *della* mutants, but not nodule development or function, may be due to a reduction in infection events or a more direct role for DELLAs in nodule initiation. The latter hypothesis is supported by the fact that *della* mutants of *Medicago* do not form nodule-like structures in transgenic roots overexpressing mutant gain of function versions of CCaMK or the cytokinin receptor *cre1* (Jin et al., 2016; Fonouni-Farde et al., 2017). Nodule-like structures were also observed in wild-type lines expressing della1-Δ18 dominant-active protein (Fonouni-Farde et al., 2017). However, several lines of evidence suggest a positive role for GA in nodule initiation and development. Firstly, an increased number of infection threads but not nodule numbers were observed in wild-type lines expressing a dominant version of *MtDELLA1* resistant to degradation by GA (Fonouni-Farde et al., 2016), suggesting a negative role for GA during infection but not necessarily nodule organogenesis. Secondly, GA-deficient *na* mutants of pea display strongly elevated numbers of infection threads, but a reduced number of nodules, and often no nodules at all (McAdam et al., 2018). Indeed, the few nodules that do form in severely GA-deficient lines are small, contain undifferentiated bacteria, and appear to fix less nitrogen than wild-type nodules (McAdam et al., 2018). This suggests an important role for GA in promoting the initiation and development of nodules into nitrogen fixing organs. This is consistent with the expression of GA biosynthesis genes within the nodule during formation and maturation in several species (Kouchi et al., 2004; Lievens et al., 2005; Hayashi et al., 2012).

Several studies have examined if this influence of GA on nodulation may be *via* interaction with other hormones with prominent roles in nodulation. In particular, studies have examined a link with ethylene, a gaseous hormone that has an overall negative influence on infection and nodule initiation and also influences the spatial arrangement of nodules (for review see Guinel, 2015). In pea, severely GA-deficient lines evolve more ethylene (Ferguson et al., 2011). To explore the interaction between GA and ethylene, double mutant lines were generated that combine the severely GA-deficient *na* mutant with *ein2*, a mutant lacking an essential element of the ethylene perception pathway (Weller et al., 2015; Foo et al., 2016). Infection, nodule initiation, and nodule function were assessed in these double mutants and compared to the respective single mutants and wild-type plants. Ethylene and GA appear to influence infection relatively independently, but GA suppression of ethylene biosynthesis may be an important mechanism to promote nodule initiation, as *na ein2* mutants develop many nodules. However, the nodules that do develop on *na ein2* plants have the characteristic arrested development of *na* (GA-deficient) single mutant plants and appear to fix little nitrogen, suggesting that GA is important for nodule development (McAdam et al., 2018). It is important to note that these *na ein2* plants still display severely reduced shoot size, suggesting that it is not shoot size *per se* that restricts nodule initiation in GA-deficient lines (Foo et al., 2016). More recently, a potential crossover between the GA and cytokinin pathways has been suggested in *Medicago* roots (Fonouni-Farde et al., 2017), although future studies are required to determine if this occurs during nodulation.

Future studies could explore the spatial and temporal regulation of GA signaling during infection and nodule development to resolve the role of GA during various stages of nodulation. This will complement ongoing studies that also indicate a role for bacterial-derived GA in some, but not all, legume-rhizobial partnerships (Tatsukami et al., 2016; Nett et al., 2017).

## Brassinosteroids

### Brassinosteroids Promote Arbuscular Mycorrhizal Symbioses

Brassinosteroids are a family of growth promoting steroid hormones. They have been implicated in a range of developmental processes, such as shoot elongation and vascular development by promoting cell elongation and division (Singh and Sigal, 2015). Only a handful of studies have investigated the role of brassinosteroids in arbuscular mycorrhizae. Overall, they appear to have a positive influence. Mutations in brassinosteroid biosynthesis genes resulting in severe BR-deficiency result in reduced arbuscular mycorrhizal colonization in tomato, rice, and pea mutants compared to wild-type plants (Bitterlich et al., 2014a,b; Foo et al., 2016) and foliar application of synthetic brassinosteroids to wheat resulted in increased in AM colonization (Tofighi et al., 2017). How BRs influences AM development is not known. As observed in the severely GA-deficient lines outlined above, severely BR-deficient lines also evolve more ethylene (Ross and Reid, 1986). Double mutant studies with ethylene insensitive plants deficient in BRs (*lk ein2*) suggest that the influence of BRs on AM is independent of ethylene (Foo et al., 2016). Other studies suggest that BRs may influence sucrose transport, potentially increasing the amount of sugar available to the fungus (Bitterlich et al., 2014a,b). Future studies are required to delineate the precise role of BRs during fungal infection, accommodation, and nutrient exchange, including any interaction with other plant hormones.

### Brassinosteroids Influence Nodulation

Most of the early studies into the role of brassinosteroids in nodulation were based on applying synthetic brassinosteroids and brassinosteroid biosynthesis inhibitors and produced mixed results, including both positive and negative effects of BR on nodulation across a range of legumes (Vardhini and Rao, 1999; Hunter, 2001; Terakado et al., 2005; Yusuf et al., 2012). This is similar to studies with GAs outlined above, where both positive and negative effects of exogenous GAs were observed on nodule number. Genetic studies using BR biosynthesis and receptor mutants in pea and a BR receptor mutant in *Medicago truncatula* suggest that BRs act as promoters of nodule number, as all the mutants form less nodules than wild type (Ferguson et al., 2005; Foo et al., 2014, 2016; Cheng et al., 2017). BRs appear to influence nodulation in part through ethylene. The elevated ethylene of severely BR-deficient pea *lk* mutant appears to explain at least in part the low nodulation phenotype of *lk* mutants, as *lk ein2* double mutants display an elevated nodulation phenotype, similar to *ein2* single mutants (Foo et al., 2016). Cheng et al. (2017) noted that the nodules that formed on the *Medicago* BR receptor mutant, *Mtbri1,* were white, suggesting that they were non-functional. However, this phenotype has not been observed in pea BR mutants (Ferguson et al., 2005). A more direct measurement of nitrogen fixation is required to determine if BR influences nodule function in addition to nodule number. It will also be interesting to examine the various stages of nodulation in BR mutants to determine if the inconsistent application results outlined above may be due to BR influencing different stages of nodule development.

It is also important to note that grafting studies in pea suggest that BR may act through a shoot-derived signal(s) to influence nodulation. Plants with reduced BR biosynthesis in the shoot system produce less nodules than those with a wild-type shoot system, irrespective of BR production in the root system (Ferguson et al., 2005). As previous grafting studies have shown that BRs are not mobile in the plant (Symons and Reid, 2004; Symons et al., 2008), BRs must be acting through a mobile signal(s). Analysis of auxin and GA levels in these grafts suggest that BR does not act *via* auxin or GA (Ferguson et al., 2005). As double mutant studies ruled out a role for BR acting upstream of the systemic autoregulation of nodulation (AON) pathway (Foo et al., 2014), future studies are required to clarify this systemic effect.

## General Conclusion

Two of the major plant growth hormone families, the GAs and BRs, influence both nodulation and arbuscular mycorrhizal symbioses. The GAs, acting through DELLAs, appear to inhibit arbuscular mycorrhizal symbioses and rhizobial infection-thread formation at least in part *via* elements of the common symbiotic pathway. GAs also appear to have a second role in nodulation, promoting nodule initiation and organogenesis. Genetic studies have to date indicated overall that BRs promote both nodulation and arbuscular mycorrhizal symbioses. However, application studies have also indicated a potentially complex role for BRs in nodulation, with both positive and negative effects. Future studies could examine how GAs and BRs influence the spatial and temporal stages of hosting symbiotic microbes, including any intersection with the common symbiotic pathway and other hormones, including those with well-defined roles in nodulation such as auxin and cytokinin.

## Author Contributions

EF conceived the project. PM, JR, and EF wrote the manuscript.

### Conflict of Interest Statement

The authors declare that the research was conducted in the absence of any commercial or financial relationships that could be construed as a potential conflict of interest.
